# The Risk of Heart Disease-Related Death Among Anaplastic Astrocytoma Patients After Chemotherapy: A SEER Population-Based Analysis

**DOI:** 10.3389/fonc.2022.870843

**Published:** 2022-06-20

**Authors:** Qi Lin, Jia-Hao Bao, Fei Xue, Jia-Jun Qin, Zhen Chen, Zhong-Rong Chen, Chao Li, Yi-Xuan Yan, Jin Fu, Zhao-Li Shen, Xian-Zhen Chen

**Affiliations:** ^1^ Department of Neurosurgery, Shanghai Tenth People’s Hospital, Tongji University School of Medicine, Shanghai, China; ^2^ Hospital of Stomatology, Guanghua School of Stomatology, Sun Yat-sen University, Guangdong Provincial Key Laboratory of Stomatology, Guangzhou, China

**Keywords:** anaplastic astrocytoma, chemotherapy, SEER, heart disease-related death, cardio-oncology

## Abstract

**Background:**

Despite improved overall survival outcomes, chemotherapy has brought concerns for heart disease–related death (HDRD) among cancer patients. The effect of chemotherapy on the risk of HDRD in anaplastic astrocytoma (AA) patients remains unclear.

**Methods:**

We obtained 7,129 AA patients from the Surveillance, Epidemiology, and End Results (SEER) database from 1975 to 2016. Kaplan–Meier and Cox regression analysis were conducted to evaluate the effect of chemotherapy on the HDRD risk. Based on the competing risk model, we calculated the cumulative incidences of HDRD and non-HDRD and performed univariate and multivariate regression analyses. Then, a 1:1 propensity score matching (PSM) was used to improve the comparability between AA patients with and without chemotherapy. Landmark analysis at 216 and 314 months was employed to minimize immortal time bias.

**Results:**

AA patients with chemotherapy were at a lower HDRD risk compared to those patients without chemotherapy (adjusted HR=0.782, 95%CI=0.736–0.83, *P*<0.001). For competing risk regression analysis, the cumulative incidence of HDRD in non-chemotherapy exceeded HDRD in the chemotherapy group (*P*<0.001) and multivariable analysis showed a lower HDRD risk in AA patients with chemotherapy (adjusted SHR=0.574, 95%CI=0.331–0.991, *P*=0.046). In the PSM-after cohort, there were no significant association between chemotherapy and the increased HDRD risk (adjusted SHR=0.595, 95%CI=0.316−1.122, *P*=0.11). Landmark analysis showed that AA patients who received chemotherapy had better heart disease–specific survival than those in the non-chemotherapy group (*P*=0.007) at the follow-up time points of 216 months. No difference was found when the follow-up time was more than 216 months.

**Conclusion:**

AA patients with chemotherapy are associated with a lower risk of HDRD compared with those without chemotherapy. Our findings may help clinicians make a decision about the management of AA patients and provide new and important evidence for applying chemotherapy in AA patients as the first-line treatment. However, more research is needed to confirm these findings and investigate the correlation of the risk of HDRD with different chemotherapy drugs and doses.

## Introduction

Gliomas are the most common primary malignant neoplasms of the central nervous system with an incidence of five-to-six cases per 100,000 persons per year. Anaplastic astrocytoma (AA), a WHO grade III glioma, is a diffusely infiltrating, malignant, astrocytic, primary brain tumor (1). It constitutes approximately 6.1% of all gliomas with a median age of onset of 41 years (2, 3). Approximately 7,175 patients were newly diagnosed from 2014 to 2018 in the United States according to the Central Brain Tumor Registry of the United States (4). Patients with AA have traditionally been thought to have a terrible prognosis. The 5-year relative survival rate for AA patients has been poor at 30.5% (95%CI=29.7–31.2), and the 10-year relative survival rate has dropped to 22.2% (95%CI=21.4–23.0) (4). Although the bulk of the tumor can often be resected, the tumor almost always reoccurs due to the rapid proliferation of infiltrative residual tumor cells (5). The standardized treatment for AA is surgical removal, radiotherapy, and chemotherapy according to the National Comprehensive Cancer Network Guidelines.

Temozolomide (TMZ), an oral monofunctional alkylating agent, which has been approved by the U.S. Food and Drug Administration (FDA) for use in the treatment of anaplastic astrocytoma for the first line, is the world-recognized standardized chemotherapy method for AA (6). Previous studies have shown that TMZ can bring overall survival benefits in AA patients and TMZ is generally better tolerated compared with the primary treatment regimen PCV (procarbazine, lomustine, and vincristine). While chemotherapy can improve overall survival outcomes, its toxicity has aroused clinicians’ and researchers’ attention. Many chemotherapy drugs such as anthracycline agents and cyclophosphamide increase the risk of cardiovascular disease, including heart failure and myocardial infarction (7). The most common toxicities of TMZ including gastrointestinal side effects and myelosuppression have been reported (8–10). Recently, TMZ has been found to be associated with liver toxicity. As for heart toxicity, the accumulation of TMZ can cause an unusual cardiomyopathy, which restricts its use in clinics (10).

However, most of the previous studies were aimed to figure out the efficacy of chemotherapy on AA patients’ overall survival, and long-term follow-up studies on the association between chemotherapy and heart disease-related death (HDRD) have been limited (8, 11–14). Thus, there is a need for clinicians and oncologists to explore whether chemotherapy increases the risk of HDRD in AA patients. The Surveillance, Epidemiology, and End Results (SEER) database provides the clinical information of cancer patients to investigate the prognostic factors of survival (15). Based on the SEER database, Guan et al. found that chemotherapy was associated with a lower cardiovascular death risk in primary central nervous system lymphoma patients than those without chemotherapy (16). Janick Weberpals found that a long-term heart-specific mortality among breast cancer survivors treated with chemotherapy or radiotherapy is not increased compared with the general population (17). However, no similar articles in AA have been published yet. Our study innovatively intends to investigate whether chemotherapy increases the risk of HDRD in AA patients on the basis of the SEER database, using competing risk regression analysis, PSM, and landmark analysis.

## Methods

### Data Source

Data were extracted from the SEER database (https://seer.cancer.gov/), which were downloaded using the SEER Stat 8.3.8 software. The SEER program is an authoritative population-based cancer registry, which covers approximately 34.6% of the U.S. population. Patients have been de-identified in the database, and no ethical approval was needed. The ethical approval of this publicly available information was not required (18).

### Study Population and Variables

Patients diagnosed with AA as a primary tumor were obtained from the SEER database. According to the International Classification of Diseases for Oncology, the Third Edition (ICD-O-3), the code of AA was 9401. We identified 7,560 patients with a diagnosis of AA between the years 1975 and 2016. Cases without a definite survival time were excluded. We also excluded patients with an unknown information of chemotherapy, and finally, 7,129 eligible patients were included for subsequent analysis.

Patients were classified into two groups depending on the chemotherapy status (yes versus no). However, the type of chemotherapy treatment and doses were unclear. Covariates included the age at diagnosis (≤34 years old, 35–50 years old, 51–65 years old, >66 years old), sex (male, female), race (white, black, others), marital status (single/unmarried, married, divorced/separated, widowed/others), year of diagnosis (~1998, 1999–2005, 2006–2012, 2013–2016), surgery (yes, no), previous history of malignant tumor (sequence number: yes, no), tumor size (≤4 cm, >4 cm, unknown), surgery method (gross total resection, subtotal resection, biopsy and local excision, no surgery, others), primary site (supratentorial tumor, infratentorial tumor, others), and radiation (yes, no). The HDRD information was extracted from the reason-of-death data from the SEER database. According to ICD-10 codes, HDRD was defined as death from heart diseases (I00–I09, I11, I13, I20–I51) including acute rheumatic fever (I00–I02), chronic rheumatic heart diseases (I05–I09), hypertensive heart disease (I11), hypertensive heart and renal disease (I13), ischemic heart diseases (I20–I25), pulmonary heart disease, the diseases of pulmonary circulation (I26–I28), and other forms of heart disease (I30–I51).

### Statistical Analysis

The different clinicopathologic characteristics between chemotherapy and non-chemotherapy groups were analyzed and evaluated using Pearson’s chi-square test. The Kaplan–Meier method was conducted to estimate heart disease-specific survival (HDSS) in different groups, and the differences between the curves were analyzed by the log-rank test. Only death from heart disease was considered as an event in the Kaplan–Meier method. For univariate and multivariate analyses, the Cox regression model was used to access the hazard ratio (HR) and 95% confidence intervals (95% CIs) to analyze the effect of chemotherapy on HDRD in AA patients. Kaplan–Meier curves and the Cox regression model were conducted using the R package “survival.”

For competing risk analysis, HDRD and other cause-related deaths were two competing endpoint events (19). HDRD was considered as the primary event of interest, whereas death due to other causes was defined as a competing risk event, and an alive patient was considered as a censored event. The probability of developing primary and competing events were shown by the calculating crude cumulative incidence function (CIF) using the Fine–Gray competing risk model, which was grouped by age, chemotherapy, diagnosis time, marital status, race, radiation, sequence number, sex, surgery, surgery method, and tumor size (20, 21). The differences in CIF among subgroups were estimated with the Gray’s test (22). The CIF curves for each variable were plotted using the R package “cmprsk”. Then, univariate and multivariate competing risk regression analyses were employed to calculate the subdistribution hazard ratio (SHR) and 95%CI to evaluate the independent effect of chemotherapy on HDRD in AA (23).

Regarding the effect of the confounding factors between the chemotherapy and non-chemotherapy groups in the SEER cohort, we employed the PSM method to improve the comparability between groups with the R package “MatchIt”. We applied age, sex, race, marital status, the year of diagnosis, primary site, tumor size, surgery, radiation, and sequence number as covariates to calculate propensity scores with a logistic regression model. The caliper value was set as 0.1. The nearest-neighbor matching method was employed, and patients were matched between 2 groups at a ratio of 1:1 (24, 25). Then, landmark analysis was conducted to avoid immortal time bias that might exist in the chemotherapy group. We chose 216 and 340 months as timepoints. The HDSS between chemotherapy and non-chemotherapy groups was estimated using the Kaplan–Meier approach. Univariate and multivariate analyses, stratified analysis, and interaction tests were also conducted in the PSM-after cohort. The landmark analysis was employed to minimize immortal time bias. Minimum follow-up times of 216 and 340 months were selected for analysis (26).

R software version 4.1.1 (https://www.r-project.org/) was used for statistical analysis and visualization (27). The following R packages were also utilized: rms, survminer, ggplot2, glmnet, pec, cobalt, and DescTools. Two-tailed *P*-values <0.05 were considered as statistically significant.

## Results

### Demographics and Clinicopathological Findings

In all, 7,129 patients diagnosed with AA were enrolled in this study. **Table 1** summarized the demographic characteristics of patients with a chemotherapy status. Of the cohort, 4,196 patients (58.9%) were stratified into the chemotherapy group, and 2,933 patients (41.1%) were stratified into the non-chemotherapy group. Statistically significant differences (*P*<0.001) were noted, between the chemotherapy group and the non-chemotherapy group, in age (42.99% vs. 57.14% age > 50 years old), marital status (26.76% vs. 24.55% single, 6.2% vs. 14.05% widowed), the year of diagnosis(61.99% vs. 32.42% after 2006), primary site (5.93% vs. 7.47% infratentorial, 75.83% vs. 70.99% supratentorial), tumor size (23.52% vs. 13.19% less than 4 cm, 21.02% vs. 9.85% more than 4 cm), surgery (59.96% vs. 34.3% yes), radiation (66.8% vs. 34.67% yes), and surgery method (16.87% vs. 10.47% biopsy and local excision, 20.97% vs. 10.64% gross total resection, 21.07% vs. 12.21% subtotal resection, 15.87% vs. 38.6% others). After a median follow-up of 75 months, there were a total of 5,257 deaths; 71 of them were related to heart disease.

**Table 1:** The demographic characteristics of anaplastic astrocytoma patients before PSM.

Characteristic	Total	Non-Chemotherapy	Chemotherapy	P	Statistic Value
	n = 7,129	n = 2,933	n = 4,196		
**Age.cat, n (%)**				<0.001	331.969
~34	1,805 (25.32)	646 (22.03)	1,159 (27.62)		
35~50	1,844 (25.87)	611 (20.83)	1,233 (29.39)		
51~65	1,795 (25.18)	664 (22.64)	1,131 (26.95)		
66~	1,685 (23.64)	1,012 (34.5)	673 (16.04)		
**Sex, n (%)**				0.039	4.265
Female	3,191 (44.76)	1,356 (46.23)	1,835 (43.73)		
Male	3,938 (55.24)	1,577 (53.77)	2,361 (56.27)		
**Race, n (%)**				0.2	3.22
Black	426 (5.98)	192 (6.55)	234 (5.58)		
Others	448 (6.28)	189 (6.44)	259 (6.17)		
White	6,255 (87.74)	2,552 (87.01)	3,703 (88.25)		
**Marital Status, n (%)**				<0.001	125.67
Divorced/Separated	518 (7.27)	213 (7.26)	305 (7.27)		
Married	4,096 (57.46)	1,588 (54.14)	2,508 (59.77)		
Single/Unmarried	1,843 (25.85)	720 (24.55)	1,123 (26.76)		
Widowed/Others	672 (9.43)	412 (14.05)	260 (6.2)		
**Diagnosis, n (%)**				<0.001	696.04
~1998	1,864 (26.15)	1,173 (39.99)	691 (16.47)		
1999~2005	1,713 (24.03)	809 (27.58)	904 (21.54)		
2006~2012	2,060 (28.9)	570 (19.43)	1,490 (35.51)		
2013~2016	1,492 (20.93)	381 (12.99)	1,111 (26.48)		
**Primary Site, n (%)**				<0.001	21.361
Infratentorial	468 (6.56)	219 (7.47)	249 (5.93)		
Others	1,397 (19.6)	632 (21.55)	765 (18.23)		
Supratentorial	5,264 (73.84)	2,082 (70.99)	3,182 (75.83)		
**Hist. Type, n (%)**				1	Fisher
Astrocytoma, anaplastic	7,129 (100)	2,933 (100)	4,196 (100)		
**Tumor Size, n (%)**				<0.001	350.623
~4	1,374 (19.27)	387 (13.19)	987 (23.52)		
4~	1,171 (16.43)	289 (9.85)	882 (21.02)		
Unknown	4,584 (64.3)	2,257 (76.95)	2,327 (55.46)		
**Surgery, n (%)**				<0.001	453.793
NO	3,607 (50.6)	1,927 (65.7)	1,680 (40.04)		
YES	3,522 (49.4)	1,006 (34.3)	2,516 (59.96)		
**Surgery Method, n (%)**				<0.001	596.69
Biopsy and local excision	1,015 (14.24)	307 (10.47)	708 (16.87)		
Gross total resection	1,192 (16.72)	312 (10.64)	880 (20.97)		
No surgery	1,882 (26.4)	824 (28.09)	1,058 (25.21)		
Others	1,798 (25.22)	1,132 (38.6)	666 (15.87)		
Subtotal resection	1,242 (17.42)	358 (12.21)	884 (21.07)		
**Radiation, n (%)**				<0.001	715.124
NO	3,309 (46.42)	1,916 (65.33)	1,393 (33.2)		
YES	3,820 (53.58)	1,017 (34.67)	2,803 (66.8)		
**Sequence Number, n (%)**				<0.001	11.381
NO	6,450 (90.48)	2,612 (89.06)	3,838 (91.47)		
YES	679 (9.52)	321 (10.94)	358 (8.53)		
**Outcome 1, n (%)**				<0.001	17.561
Non HDRD	7,058 (99)	2,886 (98.4)	4,172 (99.43)		
HDRD	71 (1)	47 (1.6)	24 (0.57)		
**Outcome 2, n (%)**				<0.001	275.38
Survival	1,872 (26.26)	473 (16.13)	1,399 (33.34)		
HDRD	71 (1)	47 (1.6)	24 (0.57)		
Competing event	5,186 (72.75)	2,413 (82.27)	2,773 (66.09)		

### Survival Analysis and Cox Regression Analysis in AA Patients

**Figure 1:**
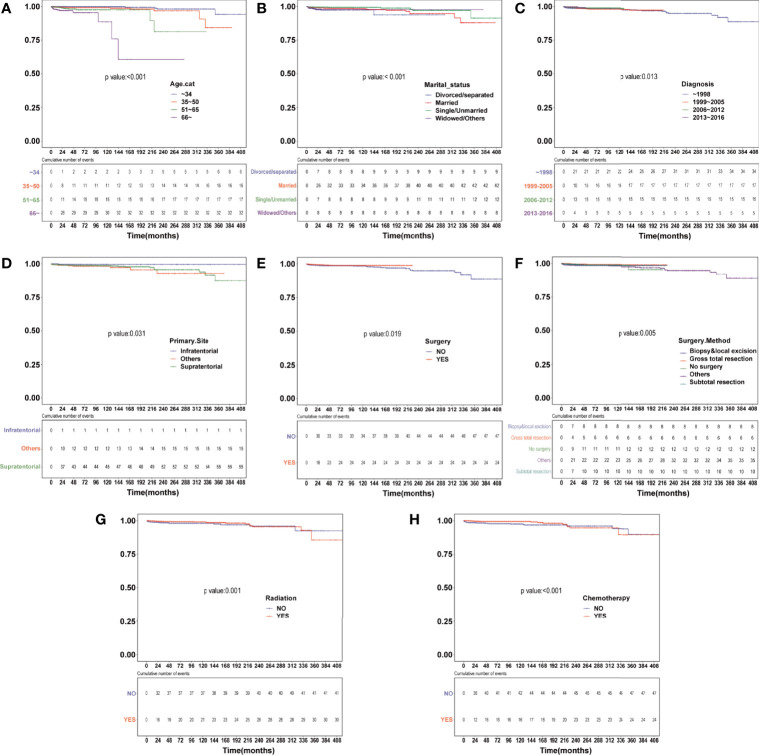
Heart disease–specific survival (HDSS) curves of anaplastic astrocytoma patients stratified according to **(A)** age at diagnosis, **(B)** marital status, **(C)** year of diagnosis, **(D)** primary site, **(E)** surgery, **(F)** surgery method, **(G)** radiation and **(H)** chemotherapy based on Kaplan–Meier method.

**Figure 2:**
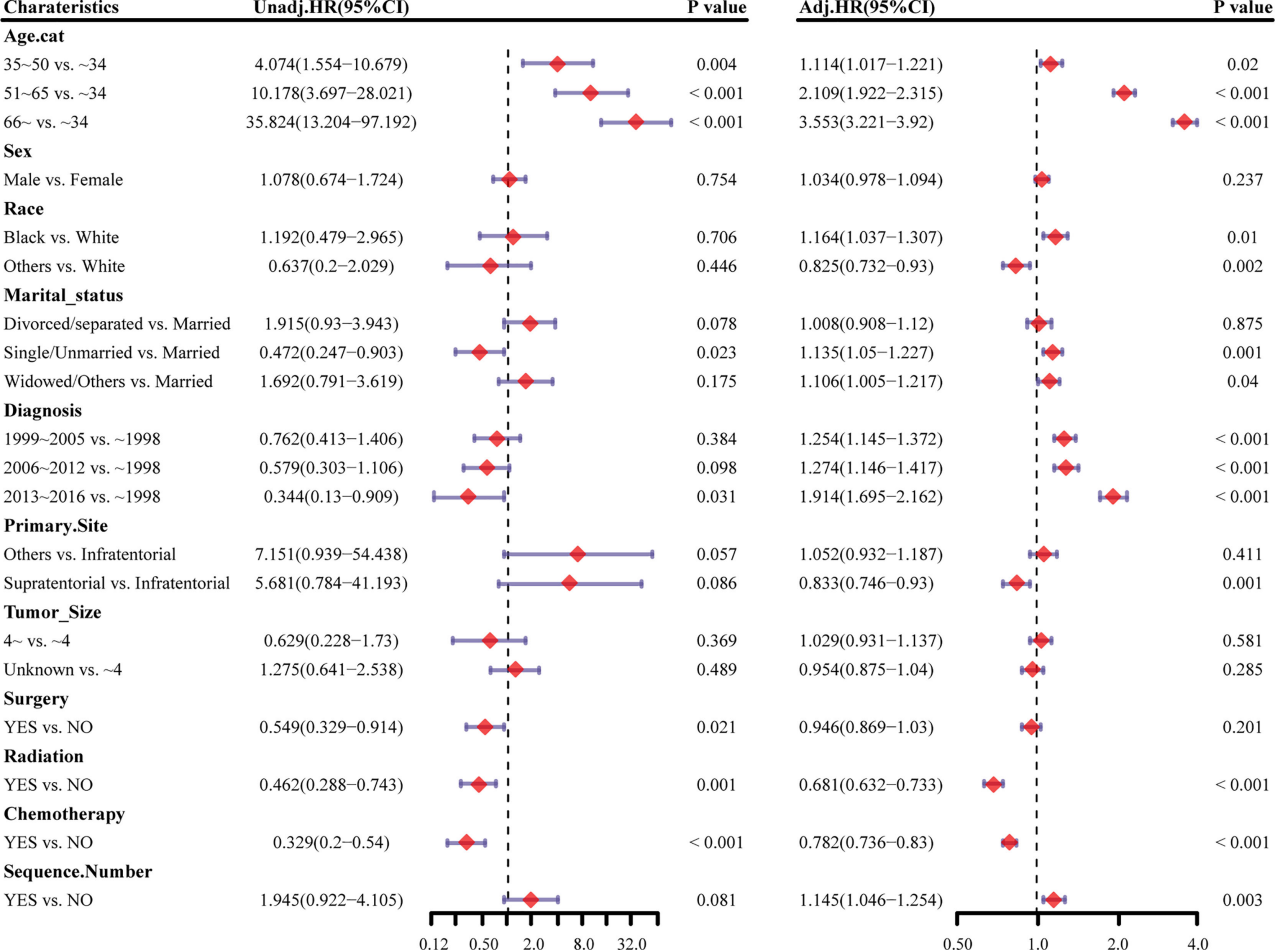
Forest plots showing different results of (left) univariable and (right) multivariable analysis for heart disease–related mortality based on the Cox proportional hazards model. HR, hazard ratio; CI, confidence interval.

Survival analysis was conducted in AA patients grouped by the chemotherapy status and covariates with a certain HDSS status and time. In the Kaplan–Meier curves and log-rank test, as shown in **Figures 1** and **S1**, patients who received chemotherapy enjoyed longer HDSS (*P*<0.001). Covariates including age at diagnosis, marital status, year of diagnosis, primary site, surgery and surgery method, and radiation were associated with HDSS. Similarly, in terms of HDSS, univariate Cox regression analysis also displayed an association between the improvement with HDSS and the patients’ chemotherapy status (unadjusted HR=0.329, 95%CI=0.2–0.54, *P*<0.001; **Figure 2**). Older age was a significant predictor of an increased HDRD. The year of diagnosis between 2013 and 2016 (unadjusted HR=0.344, 95%CI=0.13–0.909, *P*=0.031), surgery treatment in the primary tumor site (unadjusted HR=0.549, 95%CI=0.329-0.914, *P*=0.001), and radiation treatment (unadjusted HR=0.462, 95%CI=0.288-0.743, *P*=0.001) were associated with a significantly improved HDSS. After adjustment for covariates, the result of multivariate Cox regression analysis showed that the risk of HDRD was decreased in AA patients who received chemotherapy (adjusted HR=0.782, 95%CI=0.736–0.83, *P*<0.001; **Figure 2**).

### Competing Risk Regression Analysis of HDRD in AA Patients

**Figure 3:**
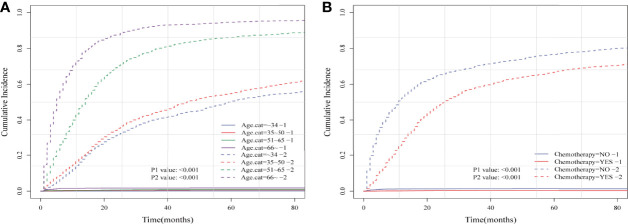
Cumulative incidence plots based on the competing risk regression model of anaplastic astrocytoma patients stratified according to **(A)** age at diagnosis and **(B)** chemotherapy status.

**Figure 4:**
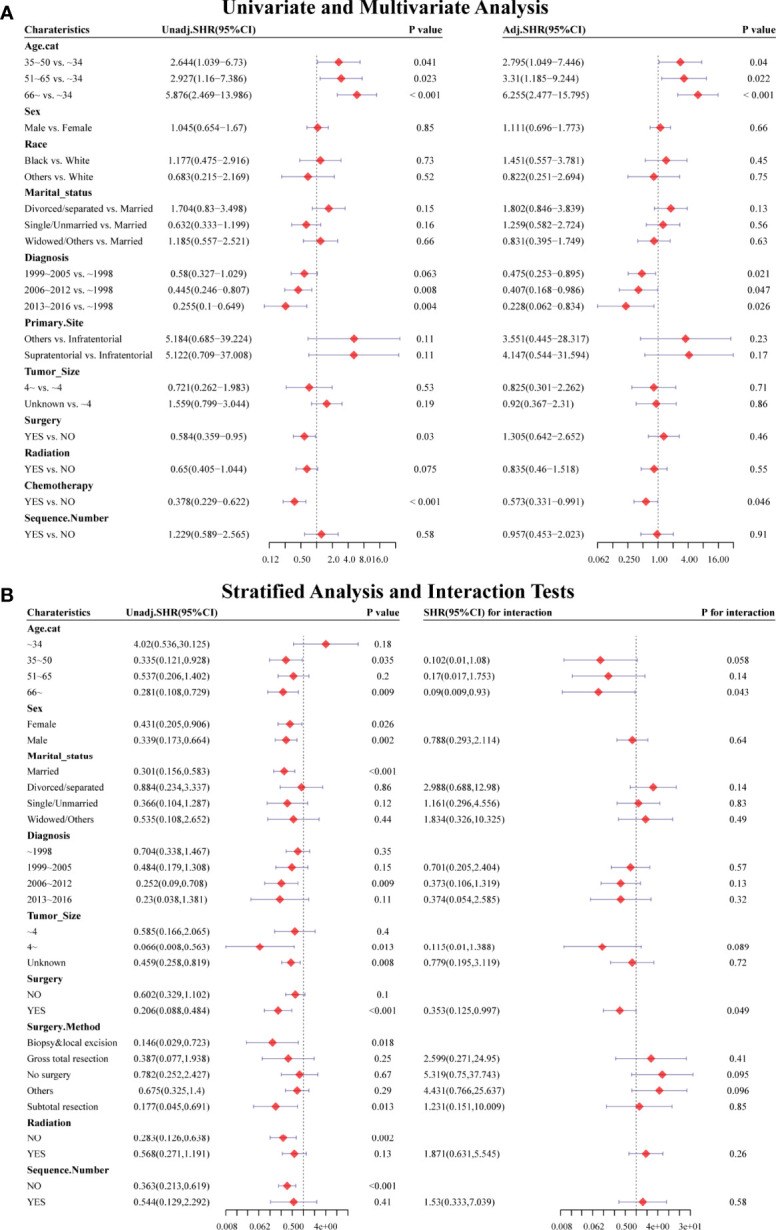
Forest plots showed the results of (**A**, left) univariable and (**A**, right) multivariable analysis based on the competing risk regression model. Forest plots showed the results of (**B**, left) stratified analysis and (**B**, right) interaction tests based on the competing risk regression model. SHR: subdistribution hazard ratio; CI, confidence interval.

Considering HRRD and other cause-related deaths were two competing endpoint events, we utilized competing risk regression analysis to explore the effect of chemotherapy on the risk of HDRD. The CIF curves for all variables are shown in **Figures 3** and **S2**. Anaplastic astrocytoma patients with chemotherapy were at a lower HDRD risk compared to those patients with no chemotherapy (*P*<0.001). Meanwhile, older age was associated with higher HDRD cumulative incidences than younger age (*P*<0.001). However, other covariates including sex, years of diagnosis, marital status, primary site, race, radiation, surgery and surgery method, sequence number, and tumor size showed no statistical significance for the cumulative incidences of HDRD. As shown in **Figure 4A**, in the competing risk regression model, univariate analysis showed the chemotherapy status (unadjusted SHR = 0.378, 95%CI = 0.229–0.622, *P*<0.001) and age (35–50 y, *P* = 0.041; 51–65 y, *P* = 0.023; >66 y, *P* < 0.001) were correlated with HDRD, which were consistent with CIF. In multivariate analysis, we adjusted covariates and found that age was independently associated with HDRD and AA patients with chemotherapy still showed a decreased probability of HDRD (adjusted SHR=0.574, 95%CI=0.331–0.991, *P*=0.046, **Figure 4A**), as expected. Additionally, we conducted stratified analysis and interaction tests to control the influence of covariates, based on several clinical factors including age, sex, marital status, year of diagnosis, tumor size, surgery and surgery method, radiation, and sequence number (**Figure 4B**). In the subgroups of age (35–50 y) and age (>60 y), patients with chemotherapy showed the decreasing risk of HDRD (35–50 y, unadjusted SHR=0.355, 95%CI=0.121–0.928, *P*=0.035; >60 y, unadjusted SHR=0.281, 95%CI=0.108–0.729, *P*=0.009). In both female and male subgroups, the married subgroup, year of diagnosis between 2006 and 2012 subgroup, tumor size >4 cm and unknown subgroup, surgery treatment in the primary site subgroup, received biopsy and local excision subgroup and subtotal resection subgroup, no radiation treatment subgroup, and no sequence number subgroup, chemotherapy was associated with a lower risk of HDRD with statistical significance. Meanwhile, chemotherapy did not increase the risk of HDRD in any of subgroups (**Figure 4B**). The subgroups of age >66 y (SHR for interaction=0.09, 95%CI=0.009–0.93, *P*=0.043) and surgery treatment in the primary site (SHR for interaction=0.353, 95%CI=0.125–0.997, *P*=0.049) displayed that there existed interactions between the effect of age/surgery and chemotherapy on the risk of HDRD (**Figure 4B**), which appeared to be effect modifiers between chemotherapy and HDRD. These results suggest robustness to our overall analysis.

### Effect of Chemotherapy on HDRD in AA Patients in PSM-After Cohort

**Figure 5:**
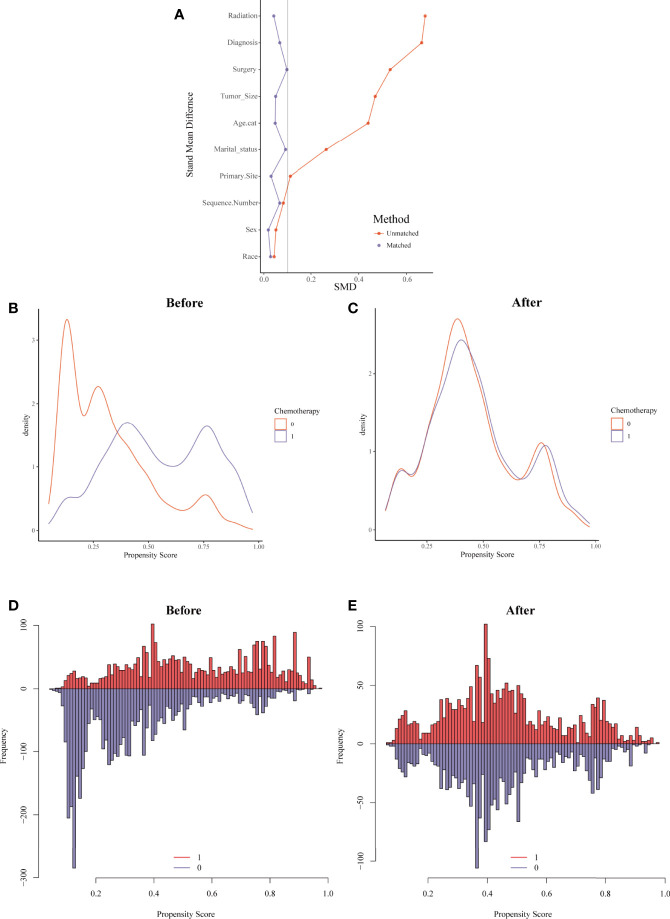
Evaluation of the covariate balance by propensity score matching (PSM). **(A)** The loveplot showed SMD across covariates before and after PSM. **(B, C)** Kernel density showed the distribution balance of chemotherapy and non-chemotherapy groups **(B)** before PSM and **(C)** after PSM. **(D, E)** Histogram showed the balance of the chemotherapy group and non-chemotherapy group **(D)** before PSM and **(E)** after PSM.

For the sake of minimizing the impact of confounding factors and confirming the role of chemotherapy on the HDRD risk, we performed a 1:1 PSM and obtained a balanced cohort (PSM-after cohort) including the non-chemotherapy group (n=2,085) and chemotherapy group (n=2,085). **Figure 5** showed the assessment methods of the covariate balance: after matching, all SMD values were lower than 0.1 (**Figure 5A**); the kernel density functions of the chemotherapy group and non-chemotherapy group were much closer than the cohort before PSM (**Figures 5B, C**); the histogram of the propensity score distribution of the chemotherapy group was similar to that of the non-chemotherapy group (**Figures 5D, E**). The characteristics of the matched patients were displayed in **Table 2**; apart from the marital status (*P* = 0.035), surgery and surgery method (*P* = 0.002), and sequence number (*P*=0.0036), almost all of the covariates were similarly distributed between the chemotherapy group and non-chemotherapy group.

**Table 2:** The demographic characteristics of anaplastic astrocytoma patients after PSM.

Characteristic	Total	Non-Chemotherapy	Chemotherapy	P
	n = 4,170	n = 2,933	n = 4,196	
**Age.cat, n (%)**				0.51
~34	1,003 (24.05)	490 (23.5)	513 (24.6)	
35~50	1,054 (25.28)	514 (24.65)	540 (25.9)	
51~65	1,069 (25.64)	548 (26.28)	521 (24.99)	
66~	1,044 (25.04)	533 (25.56)	511 (24.51)	
**Sex, n (%)**				0.575
Female	1,887 (45.25)	953 (45.71)	934 (44.8)	
Male	2,283 (54.75)	1,132 (54.29)	1,151 (55.2)	
**Race, n (%)**				0.662
Black	282 (6.76)	144 (6.91)	138 (6.62)	
Others	283 (6.79)	148 (7.1)	135 (6.47)	
White	3,605 (86.45)	1,793 (86)	1,812 (86.91)	
**Marital Status, n (%)**				0.035
Divorced/Separated	321 (7.7)	175 (8.39)	146 (7)	
Married	2,348 (56.31)	1,129 (54.15)	1,219 (58.47)	
Single/Unmarried	1,080 (25.9)	564 (27.05)	516 (24.75)	
Widowed/Others	421 (10.1)	217 (10.41)	204 (9.78)	
**Diagnosis, n (%)**				0.203
~1998	1,341 (32.16)	656 (31.46)	685 (32.85)	
1999~2005	1,142 (27.39)	561 (26.91)	581 (27.87)	
2006~2012	1,026 (24.6)	513 (24.6)	513 (24.6)	
2013~2016	661 (15.85)	355 (17.03)	306 (14.68)	
**Primary Site, n (%)**				0.629
Infratentorial	316 (7.58)	165 (7.91)	151 (7.24)	
Others	880 (21.1)	445 (21.34)	435 (20.86)	
Supratentorial	2,974 (71.32)	1,475 (70.74)	1,499 (71.89)	
**Tumor Size, n (%)**				0.285
~4	681 (16.33)	347 (16.64)	334 (16.02)	
4~	503 (12.06)	266 (12.76)	237 (11.37)	
Unknown	2,986 (71.61)	1,472 (70.6)	1,514 (72.61)	
**Surgery, n (%)**				0.002
NO	2,625 (62.95)	1,264 (60.62)	1,361 (65.28)	
YES	1,545 (37.05)	821 (39.38)	724 (34.72)	
**Surgery Method, n (%)**				0.002
Biopsy and local excision	482 (11.56)	258 (12.37)	224 (10.74)	
Gross total resection	473 (11.34)	254 (12.18)	219 (10.5)	
No surgery	1,371 (32.88)	626 (30.02)	745 (35.73)	
Others	1,292 (30.98)	659 (31.61)	633 (30.36)	
Subtotal resection	552 (13.24)	288 (13.81)	264 (12.66)	
**Radiation, n (%)**				0.192
NO	2,239 (53.69)	1,141 (54.72)	1,098 (52.66)	
YES	1,931 (46.31)	944 (45.28)	987 (47.34)	
**Sequence Number, n (%)**				0.036
NO	3,721 (89.23)	1,839 (88.2)	1,882 (90.26)	
YES	449 (10.77)	246 (11.8)	203 (9.74)	

**Figure 6:**
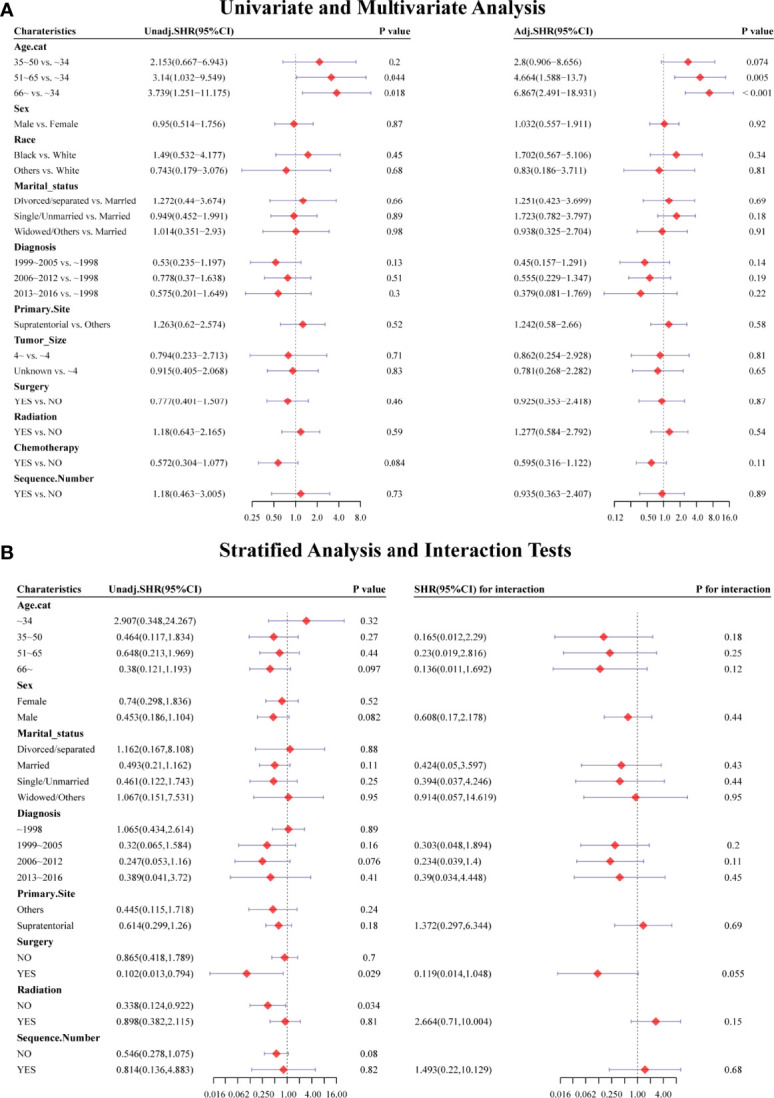
Forest plots showed the results of (**A**, left) univariable and (**A**, right) multivariable analysis in the PSM-after cohort. Forest plots showed the results of (**B**, left) stratified analysis and (**B**, right) interaction tests in the PSM-after cohort. SHR: subdistribution hazard ratio; CI, confidence interval.

Univariate and multivariate analyses based on competing risk regression analysis were conducted again in the PSM-after cohort, as shown in **Figure 6A**. The subgroup age 51–65 y (unadjusted SHR=3.14, 95%CI=1.032−9.549, *P*=0.044; adjusted SHR=4.664, 95%CI=1.588−13.7, *P*=0.005) and age >66y (unadjusted SHR=3.739, 95%CI= 1.251−11.175, *P*=0.018; adjusted SHR= 6.867, 95%CI= 2.491−18.931, *P*<0.001) were still associated with an increased probability of HDRD in both univariate and multivariate analyses. No differences were found (unadjusted SHR= 0.572, 95%CI=0.304−1.077, *P*=0.084; adjusted SHR=0.595, 95%CI=0.316−1.122, *P*=0.11) between the chemotherapy group and the non-chemotherapy group in univariate and multivariate analyses. Furthermore, we also conducted stratified analysis; the PSM-after cohort was stratified into subgroups according to covariates (**Figure 6B**). The *P*-value >0.05 appeared in almost all subgroups so that the interaction test was performed, which showed that no covariates interacted with the chemotherapy status. These results confirmed that chemotherapy for AA patients did not increase the risk of HDRD.

### Landmark Analysis of HDSS in PSM-After Cohort

**Figure 7:**
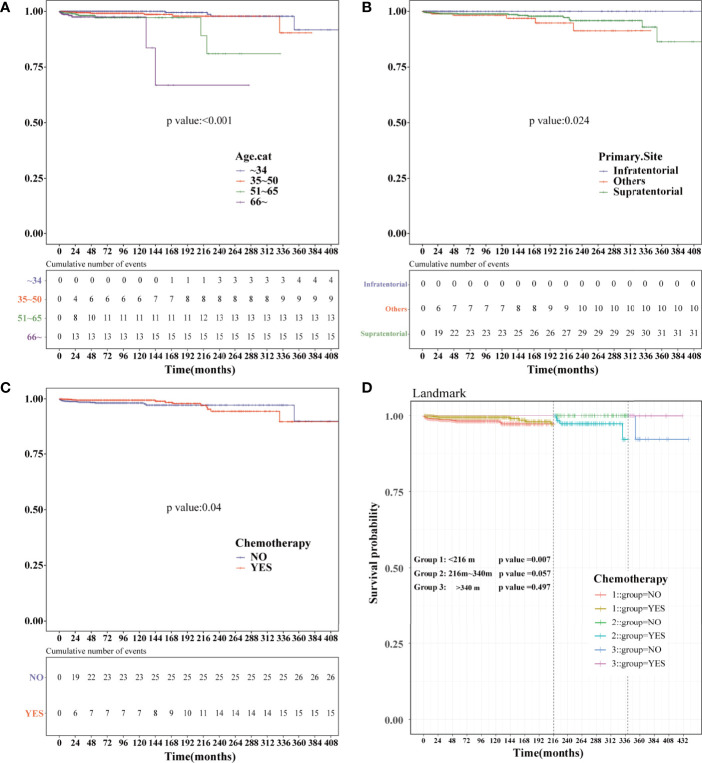
Kaplan–Meier and landmark analysis of HDSS in the PSM-after cohort. HDSS curves of anaplastic astrocytoma patients stratified according to **(A)** age at diagnosis, **(B)** primary site and **(C)** chemotherapy based on the Kaplan–Meier method. **(D)** Landmark analysis of HDSS stratified according to chemotherapy in the PSM-after cohort at 216 and 340 months.

Kaplan–Meier analysis was performed in the PSM-after cohort. The chemotherapy status (*P*=0.04), age (*P*<0.001), and primary site (*P*=0.024) were statistically significant (**Figures 7A–C**). No statistical difference was observed in sex, race, marital status, the year of diagnosis, tumor size, surgery, surgery method, radiation, and sequence number (**Figure S2**). Similar to the previous results, patients who were older or did not receive chemotherapy had a higher risk of HDRD. Subsequently, we conducted landmark analysis at 216 and 340 months to minimize immortal time bias that might exist in the chemotherapy group. Patients who received chemotherapy had better HDSS than those in the non-chemotherapy group (*P*=0.007) at the follow-up time point of 216 months. In the group of the follow-up time between 216 months and 340 months (*P*=0.057) and the group of the follow-up time of more than 340 months (*P*=0.497), there was no difference between the chemotherapy and non-chemotherapy groups in HDSS. However, information regarding chemotherapy drugs and doses was lacking. More research is needed to reveal the correlation of the risk of HDRD with different chemotherapy drugs and doses.

## Discussion

With improving long-term cancer survival, an increasing proportion of these patients are living with long-term adverse effects and complications of cancer therapy (28–30). In a previous study, Omar Abdel-Rahman found that cardiac death is a significant reason of death and there is a difference among variable cancer types (31). It was reported that cardiotoxicity is a potential adverse effect of various cancer treatments, which is responsible for significant mortality in the oncology patients, specifically due to left ventricular dysfunction (32). Many chemotherapy drugs such as anthracycline agents and cyclophosphamide improves overall survival and disease-free survival in patients in many cancer patients, but there has been an increasing concern regarding their cardiotoxicity (33, 34). These did provide an important insight into the mechanisms and principles of whether and how chemotherapy can affect cardiac function (35).

AA is a relatively rare tumor with poor prognosis (36). The optimal treatment for AA is still controversial (37). Surgery resection, radiotherapy, and chemotherapy are recommended as the first line of the treatment regimen (38, 39). Owing to the characteristics of infiltrative tumor growth, it is nearly impossible to achieve a complete surgical resection. After radiotherapy, tumor recurrence or the development of a secondary glioblastoma is usually expected. Thus, chemotherapy treatments are always recommended. Since there are limited trails about the effect of chemotherapy in AA patients, it is more necessary to understand better the risk of HDRD-associated chemotherapy in the AA patients of clinical trials (40, 41).

In this large population-based research, we utilized the SEER database to analyze the effect of chemotherapy on the risk of HDRD in AA patients. In Kaplan–Meier and Cox regression analyses, we clarified the association between clinical characteristics and HDSS and predicted the risk of an individual’s clinical outcome through HRs. We found that chemotherapy is associated with a significantly decreased risk of HDRD among AA patients. In view of existing competing events including other cause-related deaths, we conducted competing risk regression analysis to confirm the role of chemotherapy in AA patients. The result of competing risk regression analysis showed that AA patients who underwent chemotherapy were at a lower HDRD risk in comparison with those patients with no chemotherapy treatment (unadjusted SHR=0.378, 95%CI=0.229–0.622, *P*<0.001). Meanwhile, multivariate analysis confirmed the independent effect of chemotherapy on HDRD in AA (adjusted SHR=0.574, 95%CI=0.331–0.991, *P*=0.046). In addition, we found that covariates like age significantly impact on HDRD; stratified analysis and interaction tests were performed, which provided robust evidence to our analysis. Furthermore, PSM was employed between the chemotherapy group and the non-chemotherapy group to minimize the effect of covariates, in order to achieve the “post-randomization.” In the PSM-after cohort, univariate and multivariate analyses showed no significant difference between the chemotherapy group and the non-chemotherapy group on HDRD (unadjusted SHR= 0.572, 95%CI=0.304−1.077, *P*=0.084; adjusted SHR=0.595, 95%CI=0.316−1.122, *P*=0.11). Then, landmark analysis was used to correct immortal time bias in the PSM-after cohort. These results confirmed that chemotherapy did not decrease the HDSS in AA patients.

To the best of our knowledge, it is the first study to explore the effect of chemotherapy on the risk of HDRD and associated risk factors in AA patients. Since AA is a relatively rare tumor and the clinical trials were limited, the treatment regimen of AA was established according to principle of glioblastoma. The role of chemotherapy in AA, particularly TMZ, is currently under experiment (42–44). In this study, the result of our analysis identified a significantly decreased risk of HDRD with chemotherapy on AA patients, even after controlling the impact of competing events. Furthermore, both HDSS and other cause-related survival were significantly higher in AA patients with chemotherapy compared with those without chemotherapy, which may bring new insights into chemotherapy benefits in AA patients. After minimizing the effect of covariates by PSM, we still found that chemotherapy did not increase the risk of HDRD, which provided a favorable complement to our previous analysis. Moreover, patients who were diagnosed with AA decades ago were treated with no chemotherapy treatment or traditional chemotherapy regimen including the PCV regimen or carmustine, which has severe side effects such as hematologic, hepatic, and cardiac toxicity (44–46). Recently, TMZ has been widely used in AA patients with the advantages of convenience to administer, less toxicity, and similar efficacy compared to PCV (47–49). A recent randomized phase III CATION trial on concurrent and adjuvant TMZ without 1p/19q co-deletion in anaplastic glioma patients revealed an HR reduction for the overall survival of 0.645 after adjuvant TMZ (95% CI=0.450–0.926, *P*= 0.0014). Since we did not identify the impact of a specific chemotherapy treatment regimen on the risk of HDRD in AA patients, we took the year of diagnosis into consideration (50). In univariable and multivariable analyses based on the competing risk regression model, the diagnosis years between 2013 and 2016 are associated with a lower risk of HDRD compared with patients diagnosed before 1998. We supposed that it implied the impact of changes in the treatment plan, nursing treatment, and the advance of cardiovascular treatment. Nevertheless, more future clinical trials should be conducted to investigate the positive and negative roles of chemotherapy (especially TMZ) in AA patients.

In our study, we also found that age at diagnosis is one of the most important prognostic factors of HDSS and OS in AA patients, and age at diagnosis is also one of the most important covariates in our study. We employed multiple methods to minimize its impact on our main issue. Even after PSM, the older age was still associated with a high risk of HDRD in univariate and multivariate analysis, particularly in those patients who were older than 51 years old. The numbers of studies have identified the different characteristics and clinical outcomes in the malignant glioma patients of different ages (51–53). Older age is widely recognized as a risk factor and poor prognostic factor for both heart disease and cancer. In addition, the probability of receiving surgery, radiotherapy, and chemotherapy was found to be influenced by age (54–56). Elderly patients are less tolerant of the toxicity of chemotherapy drugs and more likely to suffer adverse effects and complications. Clinicians should pay more attention to the management of elder patients and should not ignore the probability of rare complications. Elderly AA patients have special needs, and a comprehensive assessment is required to provide the optimal and personalized treatment.

There are some advantages to our study. Firstly, we are the first to investigate the impact of chemotherapy on the risk of HDRD in AA patients. Secondly, due to the relatively low incidence, clinical trials were limited and difficult to perform. Simultaneously, the studies conducted in the single medical center were not applicable to discuss the risk of HDRD in cancer patients because of the small sample size, large selection bias, and low statistical efficiency. We applied the SEER database consisting of the large-scale, population-based data with longitudinal follow-up information, increasing the power to evaluate the heart disease-related outcomes. Thirdly, considering the impact of competing events, we applied the competing risk regression model to avoid false-positive results. We also utilized PSM to correct the covariables and used landmark analysis to minimize immortal time bias.

Although our study provides a robust result about the impact of chemotherapy on HDRD in AA patients, there are still some limitations. Firstly, the SEER database did not allow us to differentiate the chemotherapy information including drug regimens and doses but only “yes” versus “no/unknown” options. We cannot get access to information about specific chemotherapy drugs and regimen. Therefore, we can only assume chemotherapy drugs and give a speculation of PCV or TMZ based on the glioma treatment guidelines from the NCCN and literature reviews. Secondly, all the patients were from the United States, and the cases from Asia and Europe are still needed to verify our studies. Thirdly, as an observational study, there are some limitations in nature. Some baseline difference between groups cannot be balanced very well, compared with prospective studies, even though many methods were applied in our study. In addition, a lot of clinical information including the reason of death was missing. The exact HDRD events for each patient were not provided in the SEER database. It was suggested that if a cancer diagnosis is made very recently, death certifiers and hospital were more likely to record cancer as the cause of death. Even the rigorous quality assurance program in the SEER database (57) cannot guarantee that every medical registrar can record the exact reason of death for every cancer patient, especially those with noncancer causes. All these reasons may lead to the inaccuracy of the number of HDRD and compromise our investigation of the association between the risk of HDRD and chemotherapy in AA patients (31).

## Conclusion

In conclusion, AA patients with chemotherapy are associated with a lower risk of HDRD compared with those without chemotherapy treatment based on a large-sized population. Our findings may help clinicians make a decision about the management of AA patients and provide new and important evidence for applying chemotherapy in AA patients as the first-line treatment. Additionally, the results of our study need to be further verified in prospective randomized trials.

## Data Availability Statement

The analyzed data could be obtained at the SEER database (https://seer.cancer.gov/). Further inquiries can be directed to the corresponding authors.

## Author Contributions

X-ZC, Z-LS, and JF: study concept and design, funding acquisition, interpretation of results, article review and editing. QL and J-HB: data collection and analysis, interpretation of results, figure design, manuscript writing, article review and editing. FX, J-JQ, and Z-RC: interpretation of results and figure design. Z-RC, CL, and Y-XY: manuscript writing. All authors contributed to the article and approved the final submitted version.

## Funding

This study was supported by grants from the Project of Shenkang Hospital Development Center of Shanghai (Grant No. 16CR3048A) and the Project of Shanghai Chongming district “sustainable development science and technology” (Grant No. CKY2020-28).

## Conflict of Interest

The authors declare that the research was conducted in the absence of any commercial or financial relationships that could be construed as a potential conflict of interest.

## Publisher’s Note

All claims expressed in this article are solely those of the authors and do not necessarily represent those of their affiliated organizations, or those of the publisher, the editors and the reviewers. Any product that may be evaluated in this article, or claim that may be made by its manufacturer, is not guaranteed or endorsed by the publisher.

## Supplementary Material

Supplementary Figure 1 Heart disease specific survival curves of anaplastic astrocytoma patients stratified according to **(A)** sex, **(B)** race, **(C)** tumor size, **(D)** sequence number based on Kaplan-Meier method.

Supplementary Figure 2 Cumulative incidence plots based on competing risk regression model of anaplastic astrocytoma patients stratified according to **(A)** Sex, **(B)** race, **(C)** marital status, **(D)** the year of diagnosis, **(E)** primary site, **(F)** tumor size, **(G)** surgery, **(H)** surgery method, **(I)** radiation and **(J)** sequence number.

Supplementary Figure 3 Heart disease specific survival curves of anaplastic astrocytoma patients in PSM-after cohort stratified according to **(A)** sex, **(B)** race, **(C)** marital status, **(D)** the year of diagnosis, **(E)** tumor size, **(F)** surgery, **(G)** surgery method, **(H)** radiation and **(I)** sequence number based on Kaplan-Meier method.

## Abbreviations

AA, anaplastic astrocytoma; CI, confidence interval; CIF, cumulative incidence function; HDRD, heart disease–related death; HDSS, heart disease–specific survival; HR, hazard ratio; SEER, Surveillance, Epidemiology, and End Results; PSM, propensity score matching; SHR, subdistribution hazard ratio; TMZ, temozolomide.

## References

[1] Grimm SA Chamberlain MC. Anaplastic Astrocytoma. CNS Oncol (2016) 5(3):145–57. doi: 10.2217/cns-2016-0002 PMC6042632 27230974

[2] Louis DN Ohgaki H Wiestler OD Cavenee WK Burger PC Jouvet A. The 2007 Who Classification of Tumours of the Central Nervous System. Acta Neuropathol (2007) 114(2):97–109. doi: 10.1007/s00401-007-0243-4 17618441 PMC1929165

[3] Hong JB Roh TH Ahn SS Kim JY Kang SG Chang JH. Predicting Survival Using the 2016 World Health Organization Classification for Anaplastic Glioma. Clin Neuropathol (2020) 39(4):188–95. doi: 10.5414/np301228 32194024

[4] Ostrom QT Cioffi G Waite K Kruchko C Barnholtz-Sloan JS. Cbtrus Statistical Report: Primary Brain and Other Central Nervous System Tumors Diagnosed in the United States in 2014-2018. Neuro Oncol (2021) 23(12 Suppl 2):iii1–iii105. doi: 10.1093/neuonc/noab200 34608945 PMC8491279

[5] Wang S Yao F Lu X Li Q Su Z Lee J-H. Temozolomide Promotes Immune Escape of Gbm Cells *Via* Upregulating Pd-L1. Am J Cancer Res (2019) 9(6):1161–71. PMC6610056 31285949

[6] Jiapaer S Furuta T Tanaka S Kitabayashi T Nakada M. Potential Strategies Overcoming the Temozolomide Resistance for Glioblastoma. Neurol Med Chir (2018) 58(10):405–21. doi: 10.2176/nmc.ra.2018-0141 PMC6186761 30249919

[7] Abe J-I Yusuf SW Deswal A Herrmann J. Cardio-Oncology: Learning From the Old, Applying to the New. Front Cardiovasc Med (2020) 7:601893. doi: 10.3389/fcvm.2020.601893 33324688 PMC7723824

[8] Trinh VA Patel SP Hwu WJ. The Safety of Temozolomide in the Treatment of Malignancies. Expert Opin Drug Saf (2009) 8(4):493–9. doi: 10.1517/14740330902918281 19435405

[9] Parakh S Hamid A Cher L Gan HK. Temozolomide-Associated Liver Fibrosis. J Clin Pharmacol (2016) 56(11):1448–9. doi: 10.1002/jcph.753 27094014

[10] Huang G Zhang N Bi X Dou M. Solid Lipid Nanoparticles of Temozolomide: Potential Reduction of Cardial and Nephric Toxicity. Int J Pharm (2008) 355(1-2):314–20. doi: 10.1016/j.ijpharm.2007.12.013 18255242

[11] Nagane M. Dose-Dense Temozolomide: Is It Still Promising? Neurol Med Chir (Tokyo) (2015) 55(1):38–49. doi: 10.2176/nmc.ra.2014-0277 PMC4533399 25744349

[12] Castro GN Cayado-Gutiérrez N Zoppino FCM Fanelli MA Cuello-Carrión FD Sottile M. Effects of Temozolomide (Tmz) on the Expression and Interaction of Heat Shock Proteins (Hsps) and DNA Repair Proteins in Human Malignant Glioma Cells. Cell Stress Chaperones (2015) 20(2):253–65. doi: 10.1007/s12192-014-0537-0 PMC4326375 25155585

[13] Tan AC Ashley DM López GY Malinzak M Friedman HS Khasraw M. Management of Glioblastoma: State of the Art and Future Directions. CA Cancer J Clin (2020) 70(4):299–312. doi: 10.3322/caac.21613 32478924

[14] Zhang J Stevens MF Bradshaw TD. Temozolomide: Mechanisms of Action, Repair and Resistance. Curr Mol Pharmacol (2012) 5(1):102–14. doi: 10.2174/1874467211205010102 22122467

[15] Doll KM Rademaker A Sosa JA. Practical Guide to Surgical Data Sets: Surveillance, Epidemiology, and End Results (Seer) Database. JAMA Surg (2018) 153(6):588–9. doi: 10.1001/jamasurg.2018.0501 29617544

[16] Guan T Qiu Z Su M Yang J Tang Y Jiang Y. Cardiovascular Death Risk in Primary Central Nervous System Lymphoma Patients Treated With Chemotherapy: A Registry-Based Cohort Study. Front Oncol (2021) 11:641955. doi: 10.3389/fonc.2021.641955 34046345 PMC8147725

[17] Weberpals J Jansen L Müller OJ Brenner H. Long-Term Heart-Specific Mortality Among 347 476 Breast Cancer Patients Treated With Radiotherapy or Chemotherapy: A Registry-Based Cohort Study. Eur Heart J (2018) 39(43):3896–903. doi: 10.1093/eurheartj/ehy167 29635274

[18] Sturgeon KM Deng L Bluethmann SM Zhou S Trifiletti DM Jiang C. A Population-Based Study of Cardiovascular Disease Mortality Risk in Us Cancer Patients. Eur Heart J (2019) 40(48):3889–97. doi: 10.1093/eurheartj/ehz766 PMC6925383 31761945

[19] Dutz A Löck S. Competing Risks in Survival Data Analysis. Radiother Oncol (2019) 130:185–9. doi: 10.1016/j.radonc.2018.09.007 30314718

[20] Austin PC Fine JP. Practical Recommendations for Reporting Fine-Gray Model Analyses for Competing Risk Data. Stat Med (2017) 36(27):4391–400. doi: 10.1002/sim.7501 PMC5698744 28913837

[21] Andersen PK Keiding N. Interpretability and Importance of Functionals in Competing Risks and Multistate Models. Stat Med (2012) 31(11-12):1074–88. doi: 10.1002/sim.4385 22081496

[22] Dignam JJ Kocherginsky MN. Choice and Interpretation of Statistical Tests Used When Competing Risks Are Present. J Clin Oncol (2008) 26(24):4027–34. doi: 10.1200/JCO.2007.12.9866 PMC2654314 18711194

[23] Scrucca L Santucci A Aversa F. Regression Modeling of Competing Risk Using R: An in Depth Guide for Clinicians. Bone Marrow Transplant (2010) 45(9):1388–95. doi: 10.1038/bmt.2009.359 20062101

[24] Hwang WL Tendulkar RD Niemierko A Agrawal S Stephans KL Spratt DE. Comparison Between Adjuvant and Early-Salvage Postprostatectomy Radiotherapy for Prostate Cancer With Adverse Pathological Features. JAMA Oncol (2018) 4(5):e175230. doi: 10.1001/jamaoncol.2017.5230 29372236 PMC5885162

[25] McCaffrey DF Ridgeway G Morral AR. Propensity Score Estimation With Boosted Regression for Evaluating Causal Effects in Observational Studies. Psychol Methods (2004) 9(4):403–25. doi: 10.1037/1082-989x.9.4.403 15598095

[26] Suissa S. Immortal Time Bias in Pharmaco-Epidemiology. Am J Epidemiol (2008) 167(4):492–9. doi: 10.1093/aje/kwm324 18056625

[27] Null R Team R Null R Writing TC Null R Team R. R: A Language and Environment for Statistical Computing. Computing (2011) 1:12–21.

[28] Tykocki T Eltayeb M. Ten-Year Survival in Glioblastoma. A Systematic Review. J Clin Neurosci (2018) 54:7–13. doi: 10.1016/j.jocn.2018.05.002 29801989

[29] Ernst J Peuker M Schwarz R Fischbeck S Beutel ME. [Long-Term Survival of Adult Cancer Patients From a Psychosomatic Perspective - Literature Review and Consequences for Future Research]. Z Psychosom Med Psychother (2009) 55(4):365–81. doi: 10.13109/zptm.2009.55.4.365 20229484

[30] Stringfield O Arrington JA Johnston SK Rognin NG Peeri NC Balagurunathan Y. Multiparameter Mri Predictors of Long-Term Survival in Glioblastoma Multiforme. Tomography (2019) 5(1):135–44. doi: 10.18383/j.tom.2018.00052 PMC6403044 30854451

[31] Abdel-Rahman O. Risk of Cardiac Death Among Cancer Survivors in the United States: A Seer Database Analysis. Expert Rev Anticancer Ther (2017) 17(9):873–8. doi: 10.1080/14737140.2017.1344099 28618843

[32] Yang F Li C Guo Y Yu Y Mao S Wang R. Effects of Radical Cystectomy, Radiotherapy, and Chemotherapy on the Risk of Long-Term Heart-Specific Death in Bladder Cancer Patients. Transl Androl Urol (2021) 10(10):3826–36. doi: 10.21037/tau-21-835 PMC8575595 34804825

[33] Sparano JA. Use of Dexrazoxane and Other Strategies to Prevent Cardiomyopathy Associated With Doxorubicin-Taxane Combinations. Semin Oncol (1998) 25(4 Suppl 10):66–71. 9768827

[34] Yang R Tan C Najafi M. Cardiac Inflammation and Fibrosis Following Chemo/Radiation Therapy: Mechanisms and Therapeutic Agents. Inflammopharmacology (2021) 30(1):73–89. doi: 10.1007/s10787-021-00894-9 34813027

[35] Ananthan K Lyon AR. The Role of Biomarkers in Cardio-Oncology. J Cardiovasc Transl Res (2020) 13(3):431–50. doi: 10.1007/s12265-020-10042-3 PMC7360533 32642841

[36] Omuro A DeAngelis LM. Glioblastoma and Other Malignant Gliomas: A Clinical Review. Jama (2013) 310(17):1842–50. doi: 10.1001/jama.2013.280319 24193082

[37] Nayak L Reardon DA. High-Grade Gliomas. Continuum (Minneap Minn) (2017) 23(6, Neuro-oncology):1548–63. doi: 10.1212/con.0000000000000554 29200110

[38] Rao SAM Srinivasan S Patric IRP Hegde AS Chandramouli BA Arimappamagan A. A 16-Gene Signature Distinguishes Anaplastic Astrocytoma From Glioblastoma. PloS One (2014) 9(1):e85200. doi: 10.1371/journal.pone.0085200 24475040 PMC3901657

[39] Caccese M Padovan M D'Avella D Chioffi F Gardiman MP Berti F. Anaplastic Astrocytoma: State of the Art and Future Directions. Crit Rev Oncol/Hematol (2020) 153:7. doi: 10.1016/j.critrevonc.2020.103062 32717623

[40] Allen JC Walker R Luks E Jennings M Barfoot S Tan C. Carboplatin and Recurrent Childhood Brain Tumors. J Clin Oncol (1987) 5(3):459–63. doi: 10.1200/jco.1987.5.3.459 3546620

[41] Wick W Platten M Meisner C Felsberg J Tabatabai G Simon M. Temozolomide Chemotherapy Alone Versus Radiotherapy Alone for Malignant Astrocytoma in the Elderly: The Noa-08 Randomised, Phase 3 Trial. Lancet Oncol (2012) 13(7):707–15. doi: 10.1016/s1470-2045(12)70164-x 22578793

[42] van den Bent MJ Brandes AA Taphoorn MJ Kros JM Kouwenhoven MC Delattre JY. Adjuvant Procarbazine, Lomustine, and Vincristine Chemotherapy in Newly Diagnosed Anaplastic Oligodendroglioma: Long-Term Follow-Up of Eortc Brain Tumor Group Study 26951. J Clin Oncol (2013) 31(3):344–50. doi: 10.1200/jco.2012.43.2229 23071237

[43] Cairncross G Wang M Shaw E Jenkins R Brachman D Buckner J. Phase Iii Trial of Chemoradiotherapy for Anaplastic Oligodendroglioma: Long-Term Results of Rtog 9402. J Clin Oncol (2013) 31(3):337–43. doi: 10.1200/JCO.2012.43.2674 PMC3732012 23071247

[44] Geurts M Snijders TJ van den Bent MJ. Treatment of Anaplastic Gliomas: Evidences and Controversies. Curr Opin Oncol (2021) 33(6):621–5. doi: 10.1097/cco.0000000000000785 34456249

[45] Kristof RA Neuloh G Hans V Deckert M Urbach H Schlegel U. Combined Surgery, Radiation, and Pcv Chemotherapy for Astrocytomas Compared to Oligodendrogliomas and Oligoastrocytomas Who Grade Iii. J Neurooncol (2002) 59(3):231–7. doi: 10.1023/a:1019987116596 12241120

[46] Blondin NA Becker KP. Anaplastic Gliomas: Radiation, Chemotherapy, or Both? Hematol Oncol Clin North Am (2012) 26(4):811–23. doi: 10.1016/j.hoc.2012.04.003 22794285

[47] Brandes AA Nicolardi L Tosoni A Gardiman M Iuzzolino P Ghimenton C. Survival Following Adjuvant Pcv or Temozolomide for Anaplastic Astrocytoma. Neuro Oncol (2006) 8(3):253–60. doi: 10.1215/15228517-2006-005 PMC1871946 16723632

[48] Brada M Stenning S Gabe R Thompson LC Levy D Rampling R. Temozolomide Versus Procarbazine, Lomustine, and Vincristine in Recurrent High-Grade Glioma. J Clin Oncol (2010) 28(30):4601–8. doi: 10.1200/jco.2009.27.1932 20855843

[49] Hwang K Kim TM Park CK Chang JH Jung TY Kim JH. Concurrent and Adjuvant Temozolomide for Newly Diagnosed Grade III Gliomas Without 1p/19q Co-Deletion: A Randomized, Open-Label, Phase 2 Study (Knog-1101 Study). Cancer Res Treat (2020) 52(2):505–15. doi: 10.4143/crt.2019.421 PMC7176949 31671938

[50] Van Den Bent MJ Erridge S Vogelbaum MA Nowak AK Sanson M Brandes AA. Results of the Interim Analysis of the Eortc Randomized Phase Iii Catnon Trial on Concurrent and Adjuvant Temozolomide in Anaplastic Glioma Without 1p/19q Co-Deletion: An Intergroup Trial. J Clin Oncol (2016) 34(18_suppl):LBA2000–LBA. doi: 10.1200/JCO.2016.34.18_suppl.LBA2000

[51] Qu H-Q Jacob K Fatet S Ge B Barnett D Delattre O. Genome-Wide Profiling Using Single-Nucleotide Polymorphism Arrays Identifies Novel Chromosomal Imbalances in Pediatric Glioblastomas. Neuro Oncol (2010) 12(2):153–63. doi: 10.1093/neuonc/nop001 PMC2940568 20150382

[52] Bandopadhayay P Bergthold G London WB Goumnerova LC Morales La Madrid A Marcus KJ. Long-Term Outcome of 4,040 Children Diagnosed With Pediatric Low-Grade Gliomas: An Analysis of the Surveillance Epidemiology and End Results (Seer) Database. Pediatr Blood Cancer (2014) 61(7):1173–9. doi: 10.1002/pbc.24958 PMC4657506 24482038

[53] Jones C Perryman L Hargrave D. Paediatric and Adult Malignant Glioma: Close Relatives or Distant Cousins? Nat Rev Clin Oncol (2012) 9(7):400–13. doi: 10.1038/nrclinonc.2012.87 22641364

[54] Iwamoto FM Reiner AS Nayak L Panageas KS Elkin EB Abrey LE. Prognosis and Patterns of Care in Elderly Patients With Glioma. Cancer (2009) 115(23):5534–40. doi: 10.1002/cncr.24612 PMC7263427 19708033

[55] Iwamoto FM Cooper AR Reiner AS Nayak L Abrey LE. Glioblastoma in the Elderly: The Memorial Sloan-Kettering Cancer Center Experience (1997-2007). Cancer (2009) 115(16):3758–66. doi: 10.1002/cncr.24413 PMC7295086 19484785

[56] Smoll NR Hamilton B. Incidence and Relative Survival of Anaplastic Astrocytomas. Neuro Oncol (2014) 16(10):1400–7. doi: 10.1093/neuonc/nou053 PMC4165416 24723565

[57] Halanych JH Shuaib F Parmar G Tanikella R Howard VJ Roth DL. Agreement on Cause of Death Between Proxies, Death Certificates, and Clinician Adjudicators in the Reasons for Geographic and Racial Differences in Stroke (Regards) Study. Am J Epidemiol (2011) 173(11):1319–26. doi: 10.1093/aje/kwr033 PMC3101067 21540327

