# Corrigendum: The risk of heart disease-related death among anaplastic astrocytoma patients after chemotherapy: A SEER population-based analysis

**DOI:** 10.3389/fonc.2022.995352

**Published:** 2022-08-04

**Authors:** Qi Lin, Jia-Hao Bao, Fei Xue, Jia-Jun Qin, Zhen Chen, Zhong-Rong Chen, Chao Li, Yi-Xuan Yan, Jin Fu, Zhao-Li Shen, Xian-Zhen Chen

**Affiliations:** ^1^ Department of Neurosurgery, Shanghai Tenth People’s Hospital, Tongji University School of Medicine, Shanghai, China; ^2^ Hospital of Stomatology, Guanghua School of Stomatology, Sun Yat-sen University, Guangdong Provincial Key Laboratory of Stomatology, Guangzhou, China

**Keywords:** anaplastic astrocytoma, chemotherapy, SEER, heart disease-related death, cardio-oncology

In the published article, there was an error in affiliation 1. Instead of “^1^Department of Neurosurgery, School of Medicine, Shanghai Tenth People’s Hospital, Tongji University, Shanghai, China”, it should be “Department of Neurosurgery, Shanghai Tenth People’s Hospital, Tongji University School of Medicine, Shanghai, China” as above.

The authors apologize for this error and state that this does not change the scientific conclusions of the article in any way. The original article has been updated.

## Publisher’s note

All claims expressed in this article are solely those of the authors and do not necessarily represent those of their affiliated organizations, or those of the publisher, the editors and the reviewers. Any product that may be evaluated in this article, or claim that may be made by its manufacturer, is not guaranteed or endorsed by the publisher.

