# Supplementary figures and images for: The Risk of Heart Disease-Related Death Among Anaplastic Astrocytoma Patients After Chemotherapy: A SEER Population-Based Analysis
Source: Front Oncol. 2022 Jun 20;12:870843. doi: 10.3389/fonc.2022.870843 (PMC9251342; doi:10.3389/fonc.2022.870843)

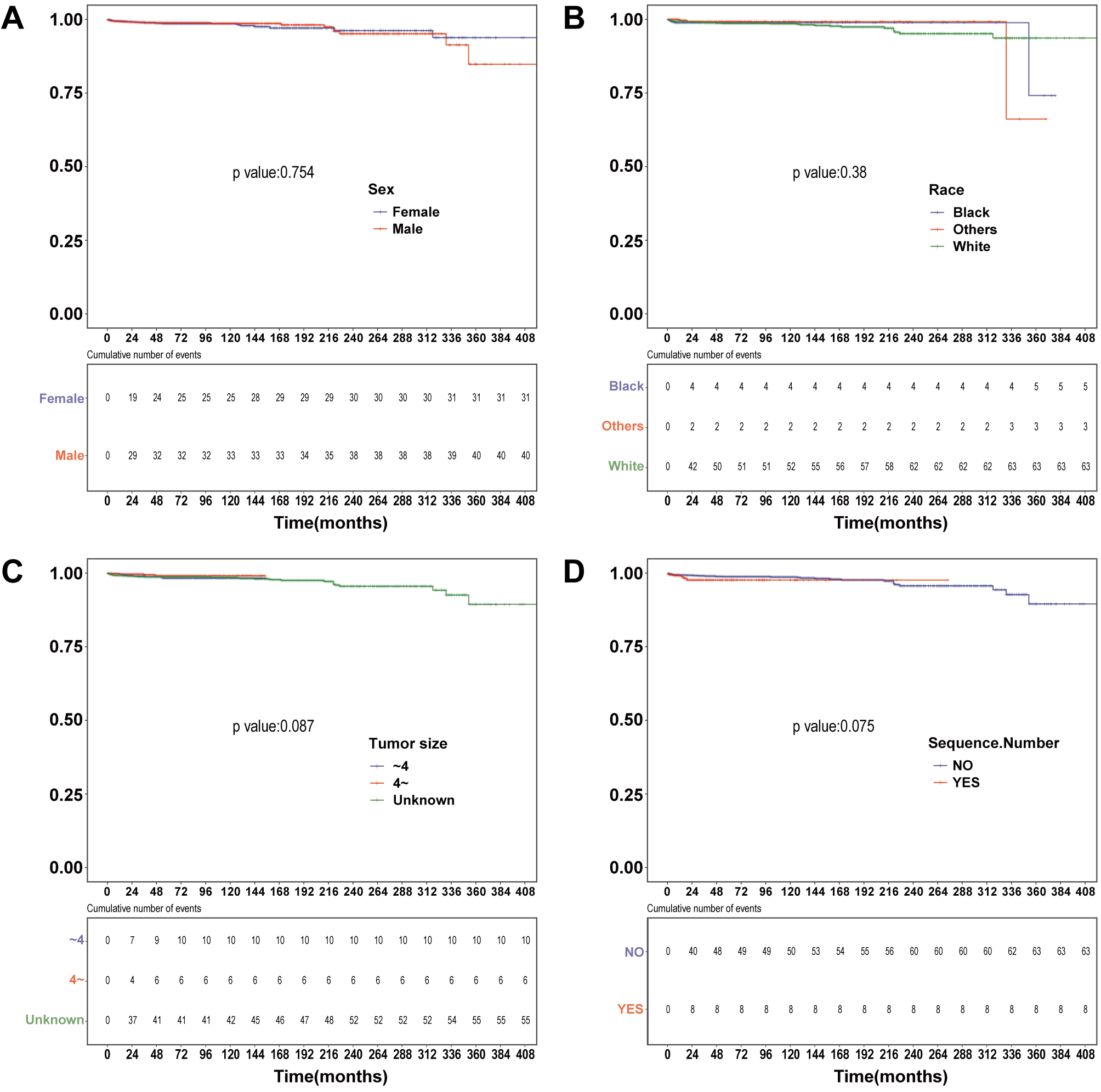

Supplement: Supplementary Figure 1 — Heart disease specific survival curves of anaplastic astrocytoma patients stratified according to (A) sex, (B) race, (C) tumor size, (D) sequence number based on Kaplan-Meier method. [file Image_1.tif]

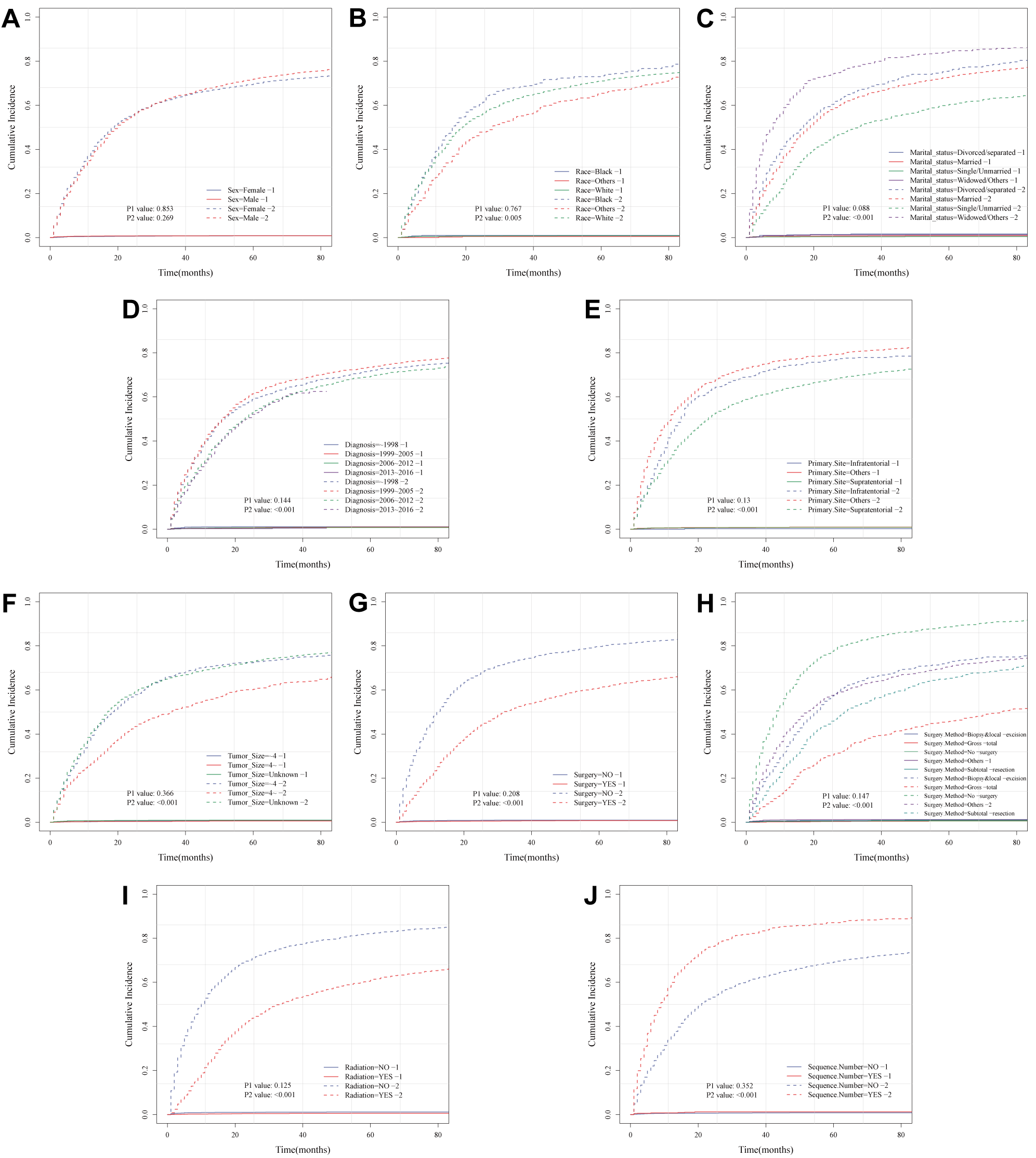

Supplement: Supplementary Figure 2 — Cumulative incidence plots based on competing risk regression model of anaplastic astrocytoma patients stratified according to (A) Sex, (B) race, (C) marital status, (D) the year of diagnosis, (E) primary site, (F) tumor size, (G) surgery, (H) surgery method, (I) radiation and (J) sequence number. [file Image_2.tif]

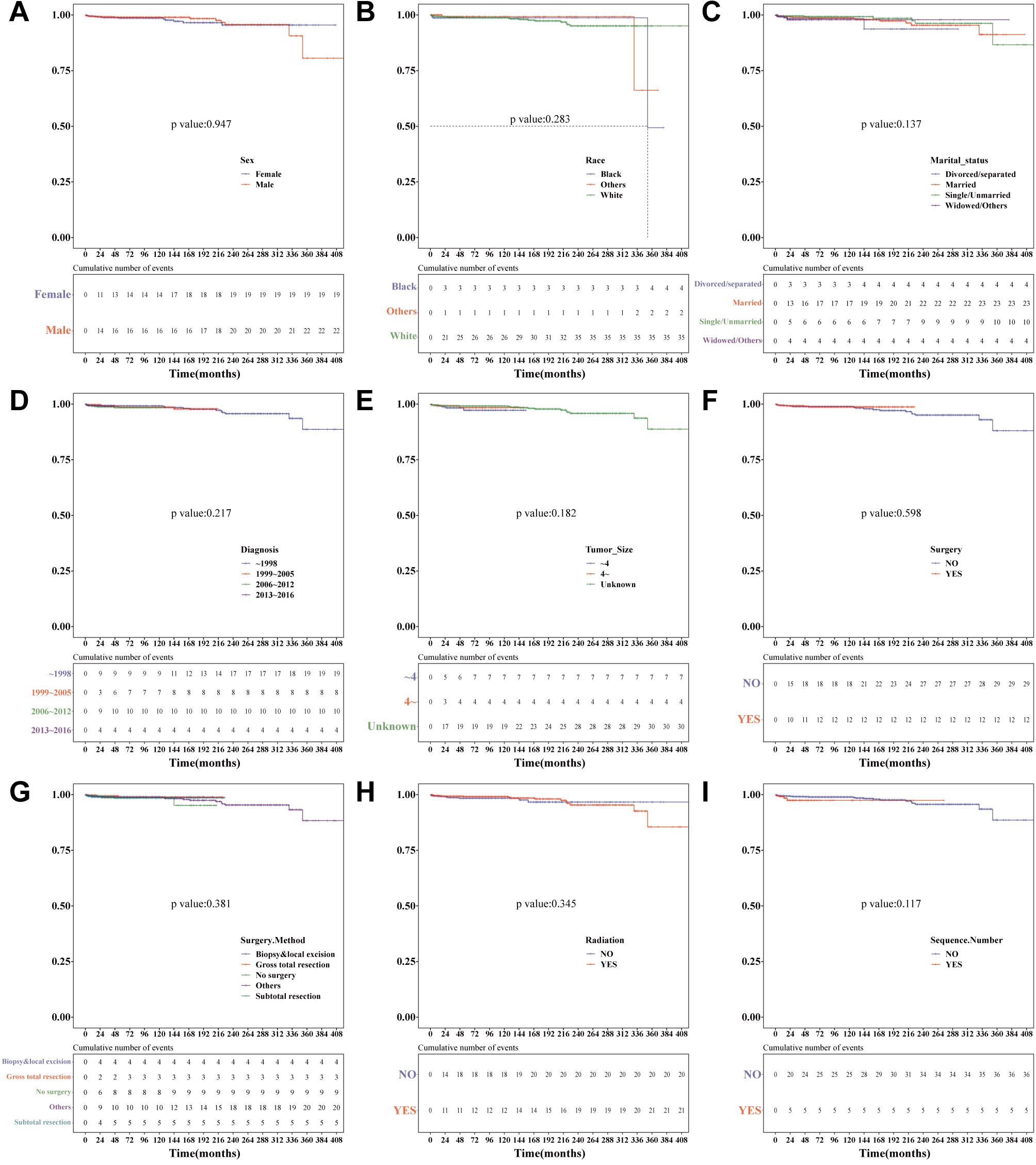

Supplement: Supplementary Figure 3 — Heart disease specific survival curves of anaplastic astrocytoma patients in PSM-after cohort stratified according to (A) sex, (B) race, (C) marital status, (D) the year of diagnosis, (E) tumor size, (F) surgery, (G) surgery method, (H) radiation and (I) sequence number based on Kaplan-Meier method. [file Image_3.tif]
